# Biaxial Yield Surface Investigation of Polymer-Matrix Composites

**DOI:** 10.3390/s130404051

**Published:** 2013-03-25

**Authors:** Junjie Ye, Yuanying Qiu, Zhi Zhai, Zhengjia He

**Affiliations:** 1 Key Laboratory of Ministry of Education for Electronic Equipment Structure Design, Xidian University, Xi'an 710071, China; E-Mail: yyqiu@mail.xidian.edu.cn; 2 State Key Laboratory for Manufacturing Systems Engineering, Xi'an Jiaotong University, Xi'an 710049, China; E-Mails: elmar@stu.xjtu.edu.cn (Z.Z.); hzj@mail.xjtu.edu.cn (Z.H.)

**Keywords:** biaxial yield surface, thermal residual stress, fiber off-axis angle, strain rate

## Abstract

This article presents a numerical technique for computing the biaxial yield surface of polymer-matrix composites with a given microstructure. Generalized Method of Cells in combination with an Improved Bodner-Partom Viscoplastic model is used to compute the inelastic deformation. The validation of presented model is proved by a fiber Bragg gratings (FBGs) strain test system through uniaxial testing under two different strain rate conditions. On this basis, the manufacturing process thermal residual stress and strain rate effect on the biaxial yield surface of composites are considered. The results show that the effect of thermal residual stress on the biaxial yield response is closely dependent on loading conditions. Moreover, biaxial yield strength tends to increase with the increasing strain rate.

## 1. Introduction

Due to their remarkable mechanical characteristics and wide range of potential applications, composites have attracted extensive attention of researchers. Composites present evident plastic behavior, which is primarily characterized by yielding and rate sensitivity in service. Because of the complexity of composite materials, experimental methods require considerable financial and human resources. Furthermore, compared with uniaxial loading conditions, it is difficult to acquire yield strength of composites through macroscopic experimental methods under complex stress conditions. Therefore, more and more investigators rely to theoretical research on biaxial yield responses of composites.

Two main methods, namely the analytical micromechanical method and the finite element method, have been used to study yield the behaviors of composites under complex stress conditions. Azizi *et al.* [1] utilized a finite element method to study the size-effects on initial yield surfaces, and subsequent yield surfaces for reinforced composites under axial-torsion loading conditions. Tang and Yu [2] adopted a finite element method to predict the initial yielding surface of heterogeneous materials with realistic microstructures. Moshtaghin *et al.* [3] constructed a micromechanical method to investigate the effects of surface residual stress as well as surface elasticity on the overall yield strength of nanoporous metal matrices containing aligned cylindrical nanovoids. Acton and Graham [4,5] used a moving window Generalized Method of Cells to approximate a yield surface. Moreover, in order to determine the accuracy of the models, each result was compared with an analytical study. Through application of the Mean-field Homogenization method, Selmi *et al.* [6] predicted the biaxial yield behavior, hardening and plastic flow of misaligned short fiber-reinforced composites. For laminated metal matrix composites, Radi and Abdul [7] described the evolution of the yield surface using a recent developed self-consistent model with small strains assumption. Lissenden [8,9] using a proof strain criterion for the permanent strain that relies on cyclic, proportional to probe the *loci* of the yield surface. Furthermore, initial and subsequent yield surfaces of highly anisotropic materials were studied by experimental methods. However, few studies concerning the thermal residual stress and strain rate influence on the yield surface of composites with different fiber off-axis angles have been reported.

In addition, it should be noted that traditional strain gauges can hardly capture dynamic strain changes exactly under high-rate loading conditions due to the sensitivity to electromagnetic interference and low speed response. In this paper, repeatability and sensitivity of FBGs sensor are validated by a cantilever system. Meanwhile, the prediction results under uniaxial tensile conditions are validated by experimental data of a FBGs strain test system. On this basis, the effects of thermal residual stress and strain rate on the yield surfaces of composites with different fiber off-axis angles are investigated.

## 2. Micro-Mechanical Models of Fiber-Reinforced Composites

### 2.1. Representative Volume Element

**Figure 1.**
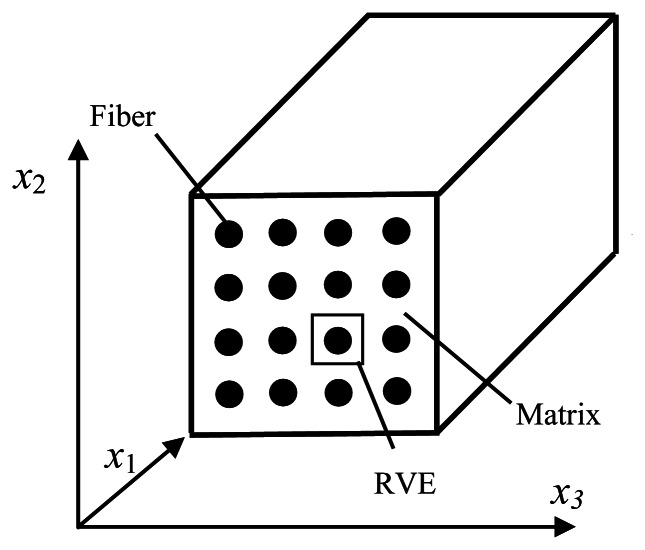
Fiber-reinforced composites with periodic array.

In the most micro-mechanical models, it is supposed that inclusions or fibers present periodic configuration in the composites, as shown in Figure 1. Through choosing a proper unit, a micro-mechanical constitutive model of the composites can be established. On this basis, macro-mechanical behaviors can be acquired from the homogenization theory.

### 2.2. Generalized Method of Cells

**Figure 2.**
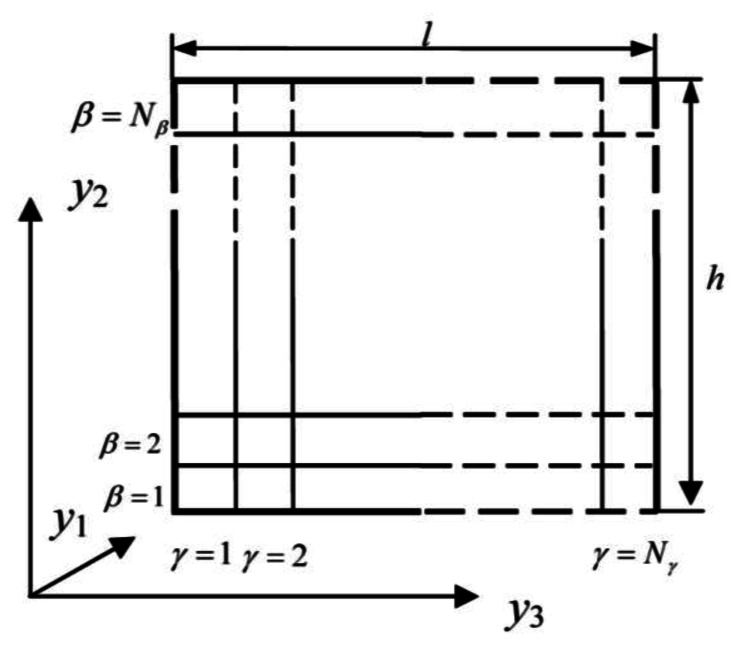
Discretization of the RVE.

Generalized Method of Cells (GMC), one of the most important micromechanical models, has been used in predicting effective elastic constants, mechanical properties of composites [10, 11 and 12]. For fiber-reinforced composites, the representative volume element (RVE) is extracted from the cross section which is perpendicular to the fiber direction. The RVE is divided into *N_β_* × *N_γ_* sub-cells as shown in Figure 2. In the figure, *h* and *l* indicate the length of the RVE in the *y*_2_ and *y*_3_ directions, respectively. *β* and *γ* indicate the number of the sub-cells in the *y*_2_ and *y*_3_ directions, respectively. The constitutive equation of sub-cells is given by:

(1) $${\bar{\sigma }}^{\left(\beta \gamma \right)}=C^{\left(\beta \gamma \right)}\;({\bar{ɛ}}^{\left(\beta \gamma \right)}-{\bar{ɛ}}^{P(\beta \gamma )}-\alpha^{\left(\beta \gamma \right)}\Delta T)$$

where *σ̅* ^(*βγ*)^ is the average stress of the sub-cells. *C*^(*βγ*)^ is the stiffness matrix of the sub-cells. *ε̅*^(*βγ*)^ and *ε̅*^-p(*βγ*)^ indicate the average strain and plastic strain of the sub-cells. *α*^(*βγ*)^ and Δ*T* indicate thermal expansion coefficient of the sub-cells and temperature change.

According to the homogenization theory, the relationship between macroscopic average stress *σ̅* and sub-cell average stress *σ̅*^(*βγ*)^ can be expressed as:

(2) $$\begin{array}{ll} \bar{\sigma } & =\frac{1}{hl}\;\overset{N_{\beta }}{\underset{h=1}{\text{∑}}}\;\overset{N_{\gamma }}{\underset{\gamma =1}{\text{∑}}}{\bar{\sigma }}^{\left(\beta \gamma \right)}\\ & =C^{*}(\bar{ɛ}-{\bar{ɛ}}^{p}-\alpha \Delta T) \end{array}$$

where C* is macroscopic stiffness of composites, *ε̅* and *ε̅*^p^ indicate the macroscopic average strain and plastic strain, respectively. *α* is macroscopic thermal expansion coefficient.

In order to satisfy displacement continuity conditions between adjacent sub-cells and axial deformation constraint conditions, the relationship between sub-cell average strain and macro strain can be expressed as follows:

(3) $$A_{G}{\bar{ɛ}}_{s}=J\bar{ɛ}$$

where *A_G_* contains geometric dimension of the sub-cells. *J* contains the geometric dimension of the RVE. *ε̅* indicates strain vector of the sub-cells. *ε̅* is the component of macro-strain.

According to the stress continuity conditions between sub-cells and the constitutive equation of the sub-cells, the relationship among average plastic strain components ${\bar{ɛ}}_{S}^{P}$, average thermal strain components ${\bar{ɛ}}_{S}^{T}$, and average strain components *ε̅_S_* can be established as follows:

(4) $$A_{M}({\bar{ɛ}}_{s}-{\bar{ɛ}}_{s}^{p}-{\bar{ɛ}}_{s}^{T})=0$$

where *A_M_* contains the stiffness matrix of the sub-cells.

Combining Equations (3) and (4), the sub-cells strain vector *ε̅_S_* can be acquired. Furthermore, average sub-cell strains *ε̅*^(*βγ*)^ can be expressed as:

(5) $${\bar{ɛ}}^{\left(\beta \gamma \right)}=A^{\left(\beta \gamma \right)}\bar{ɛ}+D^{\left(\beta \gamma \right)}({\bar{ɛ}}_{S}^{P}+{\bar{ɛ}}_{S}^{T})$$

where concentrations matrices *A*^(*βγ*)^ refers to stiffness matrix as well as geometric dimensions of the sub-cells. *D*^(*βγ*)^ refers to the elastic constants of the sub-cell materials.

Substituting Equation (5) into Equation (1), sub-cell average stress *σ̅*^(*βγ*)^ can be acquired. On this basis, substituting *σ̅*^(*βγ*)^ into Equation (2), macroscopic stress *σ̅* can be written as:

(6) $$\bar{\sigma }=\frac{1}{hl}\sum_{\beta =1}^{N_{\beta }}\sum_{\gamma =1}^{N_{\gamma }}h_{\beta }l_{\gamma }C^{\left(\beta \gamma \right)}\left[A^{\left(\beta \gamma \right)}\bar{ɛ}+D^{\left(\beta \gamma \right)}({\bar{ɛ}}_{S}^{P}+{\bar{ɛ}}_{S}^{T})-({\bar{ɛ}}_{S}^{P(\beta \gamma )}+{\bar{ɛ}}_{S}^{T(\beta \gamma )})\right]$$

Through comparing Equation (6) with Equation (2), macroscopic stiffness matrix C* can be expressed as follows:

(7) $$C^{*}=\frac{1}{hl}\sum_{\beta =1}^{N_{\beta }}\sum_{\gamma =1}^{N_{\gamma }}h_{\beta }l_{\gamma }C^{\left(\beta \gamma \right)}A^{\left(\beta \gamma \right)}$$

## 3. Experimental Verification

### 3.1. Experimental Research on a FBGs Sensor

**Figure 3.**
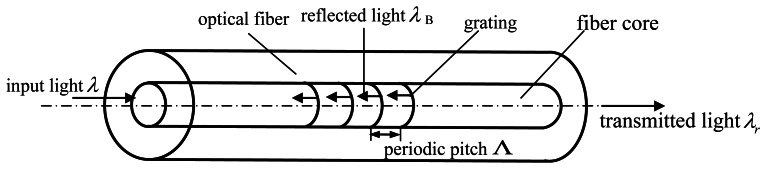
The structure of FBGs sensor.

Due to their small dimensions and accurate measurements, as well as resistance to corrosion and electromagnetism, FBGs sensors have been used in cardiac ablation [13], structural health monitoring [14,15], as well as the biomechanics and rehabilitation fields [16]. The structure of a FBGs sensor can be seen in Figure 3. It is composed of an optical fiber, grating and fiber core. The fabrication process of FBGs sensor is due to the photosensitivity property of the doped silica glass fiber core. A permanent grating can be inscribed into the photosensitive fiber core when exposed to ultraviolet light and is usually obtained by means of the two-beam interference technique or phase mask method. The principle of the FBGs sensor is to measure the changes of center wavelengths of reflective light from a Bragg grating. With the variation of strain, the center wavelengths of the reflection light will be corresponding changed. The Bragg wavelength can be expressed as follows [17]:

(8) $$\lambda_{\beta }=2n_{eff}\Lambda$$

where *n_eff_* is the effective reflective index of the fiber core, Λ is the grating periodic spacing, *λ_β_* is the wavelength of reflected light. It can be found from Equation (8) that the Bragg wavelength will shift with the parameter of *n_eff_* and Λ. Disregarding the thermal influence, the periodic spacing and effective reflective index will change when the mechanical deformation is posed on the grating area. The relationship between Bragg wavelength shift and the change of strain (Δ*ε*) can be expressed as:

(9) $$\Delta \lambda_{B}=\alpha \Delta ɛ$$

where:

(10) $$\alpha =\lambda_{B}(1-p_{e})$$

and *p_e_* is an effective strain-optic constant.

In acquiring strain signals, the next two methods are always used [18]: directly pasted on surface or embedded into structures. In this paper, the former method is used to test the strains of composites. Before making use of the FBGs sensor, repeatability and sensitivity experiments are performed. In the experiment, a SM130-700 fiber grating demodulator, which is produced by Micron Optical International Corporation, is used to measure optical signal. The parameter of SM130-700 is as follows: Wavelength scanning range is 1,510–1,590 nm. Resolution is 1 pm. Scanning frequency is 1,000 Hz.

#### 3.1.1. Repeatability Experiment

**Figure 4.**
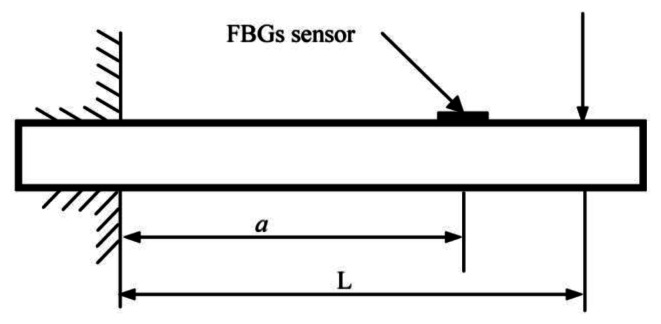
Test principle diagram.

In order to validate the strain test of FBGs sensor, a cantilever beam structure is used as shown in Figure 4. The corresponding parameters are as follows: the width and height of the cantilever beam are 12 mm and 20 mm, respectively. Elastic modulus is 206 Gpa. The parameter *a* = 424 mm indicates the distance between central position of FBGs sensor and the fixed end of the cantilever beam. The parameter *L* = 469 mm indicates the distance between the loading position and the fixed end of cantilever beam. Loading sequence is as follows: 19.6N, 39.2N, 49.0N, 58.8N, 68.6N. The relationship between measuring wavelength *A_i_* of FBGs sensor and measuring strain *ε* can be written as follows [19]:

(11) $$ɛ=(A_{i}-A_{0})*1000/1.2$$

where *A*_0_ indicate initial wavelength. The parameter 1,000 converts the Bragg wavelength shift from nm to pm. The parameter 1.2 indicates the strain sensitivity of the FBGs sensor, which is provided by Micron Optical International Corporation.

**Figure 5.**
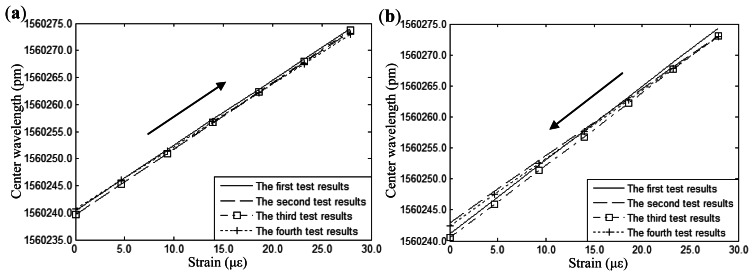
Repeatability experiment of FBGs sensor: (**a**) Loading conditions. (**b**) Unloading conditions.

**Figure 6.**
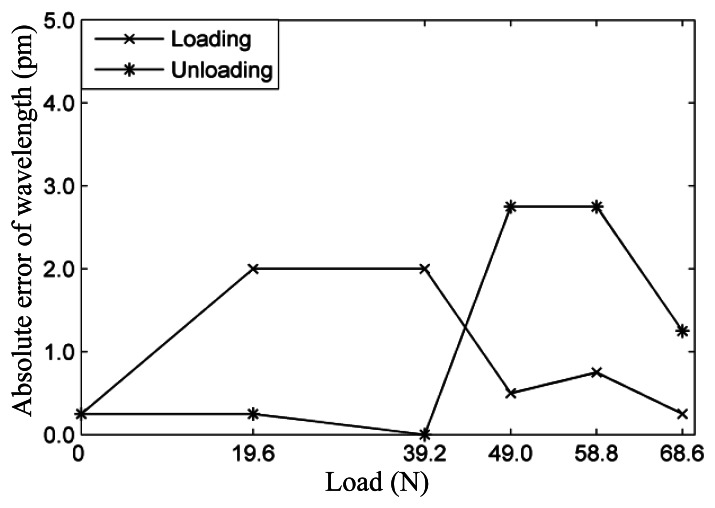
Wavelength absolute error of the FBGs sensor under loading and unloading conditions.

Measurement data are linear fitted by the least-squares method. Figure 5(a,b) shows the experimental results under loading and unloading conditions, respectively. It can be seen that measured results of the FBGs sensor show high reproducibility under loading and unloading conditions. Figure 6 shows the wavelength absolute error of the FBGs sensor between the theoretical wavelength and average wavelength of the test results. It can be seen from the figure that absolute error is less than 3 pm.

#### 3.1.2. Sensitivity Experiment

**Figure 7.**
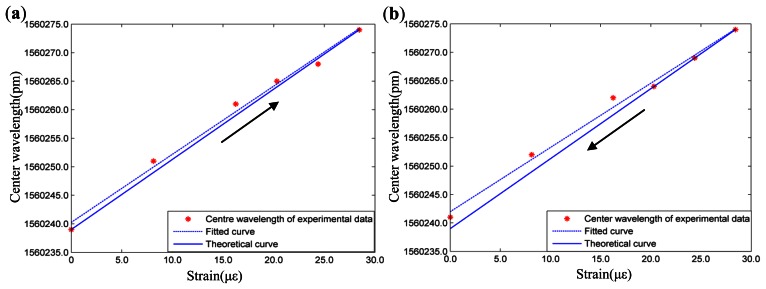
Sensitivity experiment of the FBGs sensor: (**a**) Loading conditions. (**b**) Unloading conditions.

The average wavelengths of four repeated tests are used to fit the strain sensitivity coefficients by the least-squares method under loading and unloading conditions. The sensitivity experimental results and corresponding error analysis can be seen in Figure 7 and Table 1, respectively. Moreover, the theoretical result of the strain sensitivity coefficient (1.200), which can be acquired from Equation (11), are also shown in the figures. From the Table 1, it can be seen that the strain sensitivity coefficients of the FBGs sensor under loading and unloading conditions are 1.194 and 1.158, respectively. Comparing with the theoretical results, the relative errors of the experimental results are 0.50% and 3.50%, respectively. In addition, correlation coefficients of the theoretical curve and the fitted curve under conditions of loading and unloading are 0.9959 and 0.9948, respectively. Based on the studies mentioned above, it can be seen that the FBGs sensor directly pasted on the structure presents high selectivity in strain tests.

### 3.2. Experimental Research on Micromechanical Model

In order to describe the nonlinear behaviors of polymer matrix composites, an Improved Bodner-Partom (IBP) model is incorporated into the GMC model. Supposing that the fiber is linearly elastic, the polymer matrix is viscoplastic. The flow law for the viscoplastic strain rate components of the IBP model is formulated as follows [20]:

(12) $${\overset{\cdot }{ɛ}}_{ij}^{I}=2D_{0}\exp\left[-\frac{1}{2}{\left(\frac{Z}{\sigma_{e}}\right)}^{2n}\right]\;(\frac{S_{ij}}{2\sqrt{J_{2}}}+\alpha \delta_{ij})$$

where

$$\begin{array}{c} J_{2}=\frac{1}{2}s_{ij}s_{ij}\dot{Z}=q(Z_{1}-Z){\dot{e}}_{e}^{I}\dot{\alpha }=q(\alpha_{1}-\alpha ){\dot{e}}_{e}^{I}\\ {\dot{e}}_{e}^{I}=\sqrt{\frac{2}{3}{\dot{e}}_{ij}^{I}{\dot{e}}_{ij}^{I}}{\dot{e}}_{ij}^{I}={\dot{ɛ}}_{ij}^{I}-{\dot{ɛ}}_{m}^{I}\delta_{ij} \end{array}$$

In the above, the overhead dot of the variables indicates the differentiation with respect to time *t*. Material parameters *n*, *Z*_0_, *Z*_1_, *α*_0_, *α*_1_ and *q*, which can be acquired by axial tension and pure shear experimental tests, are referred to as hardening characteristics. Six material parameters of the polymer matrix mentioned above can be determined through tensile and shear tests. According to the ASTM D3039 test, the dimension of the polymer specimen is 250 × 25 × 3 mm. According to the ASTM D5379, the length and width of polymer specimen is 76.2 × 19.1 mm, while the thickness of the polymer specimen can be determined as required. In the experiment, uniaxial tension tests with strain rate 10^−5^/s and pure shear tests with strain rates 10^−5^/s and 10^−1^/s are used to determine the six material parameters. The details can be seen in the reference [21].

**Figure 8.**
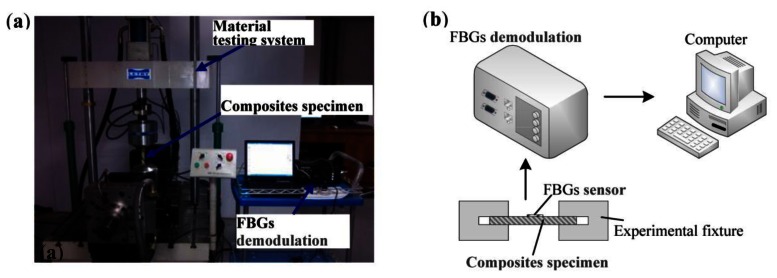
Experimental system of the FBGs strain test system: (**a**) Picture. (**b**) Schematic.

**Figure 9.**
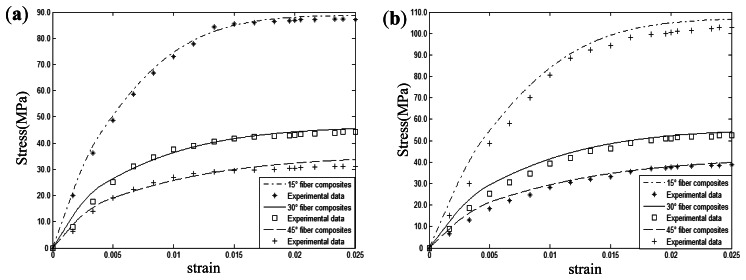
Experimental research on polymer-matrix composites: (**a**) Strain rate 0.00001/s. (**b**) Strain rate 0.01/s.

Figure 8(a,b) shows the picture and schematic of experimental system. The test system is made up of a Fiber Bragg Gratings (FBGs) sensor, composites specimen, material testing system and FBGs demodulation devices. Aluminum alloy tabs of 1 mm thickness were attached to the two ends of the specimen. The elastic modulus, Poisson's ration as well as viscoplastic parameters can be seen in Table 2. To validate the presented model, the uniaxial tensile mechanical responses of 15°, 30°, 45° fiber-reinforced composites with 0.24 fiber volume fraction were measured under two different strain rate (0.00001/s, 0.01/s) conditions. Theoretical results and experimental data are shown in Figure 9. It can be seen that theoretical prediction in different strain rate conditions shows excellent agreement with the experimental results. Comparing Figure 9(a) with Figure 9(b), it can be easily found that increasing strain rate will increase the yield strength of composites under uniaxial tension. For instance, compared with the strain rate 0.00001/s, polymer-matrix composites provide a stress at the 2.5% strain that is approximately 20% higher than the stress of the composites with a strain rate of 0.01/s under 15° fiber off-axis angle conditions.

## 4. GMC-Based Prediction of Biaxial Yield Response

Good correlation has been found between the micromechanical model and the experimental data. On this basis, the biaxial yield responses of polymer-matrix composites are studied.

### 4.1. Yield Surface Definitions

With the increase of loading, material deformation is translated from the elastic to the plastic state. This procedure is called yield. A yield surface is defined as the locus of points in a stress or strain space when a specified yield criterion is satisfied. Stress state of materials can be easily discerned by the stress yield surface. Generally speaking, the relationship between stress *σ_ij_*, strain *ε_ij_*, time *t* as well as temperture *T* and yield function Φ can be written as:

(13) $$\Phi (\sigma_{ij},{ɛ}_{ij},t,T)=0$$

Under the condition of ignoring parameters *t* and *T*, yield function is related to stress and strain of the materials. In the stress space, when the load stress cannot reach the yield surface, the materials can be considered to be in an elastic state. Once stress lies on the yield surface, the materials begin to yield.

Many researchers have studied the yield surface of materials by experimental methods. The results show that the yield surface presents different shapes. Ishikawa [22] indicated that subsequent yield surface presents elliptic shapes without a shape corner and cross effect. However, Kan *et al.* [23,24] pointed out that a sharp corner in preloading direction and cross effect on the normal preloading direction in condition of complicated loading can be observed. The different results derive from different definition of yield [25,26]. At present, three important yield criteria for composites can be expressed as follows [6,27]:

(1) $\sqrt{{\dot{ɛ}}_{ij}^{I}{\dot{ɛ}}_{ij}^{I}}$ is defined as surfaces of constant inelastic strain rate (SCISRs)
(2) ${\bar{\sigma }}_{ij}{\dot{ɛ}}_{ij}^{I}$ is defined as surfaces of constant inelastic power (SCIPs)
(3) ${ɛ}_{ps}=\sqrt{\frac{2}{3}{ɛ}_{ij}^{I}{ɛ}_{ij}^{I}}$ is defined as surfaces of equivalent plastic strain (SEPSs)

where σ̅*_ij_* refers to average stress. *ε_ps_* is equivalent plastic strain. ${\dot{ɛ}}_{ij}^{I}$ and ${ɛ}_{ij}^{I}$ indicate inelastic strain rate and inelastic strain of the materials.

In order to build a fiber-reinforced composites yield surface, equivalent plastic strain *ε_ps_* = 0.001 is used to investigate the biaxial yield response of composites in this paper. For each condition, biaxial yield responses are mapped out by a constant strain ratio, namely, *ε_xx_*/*ε_yy_* = constant or *ε_xx_*/*ε_xy_* = constant.

### 4.2. Thermal Residual Stress and Strain Rate Influence on Biaxial Yield Response

As mentioned in references [28,29], uniaxial inelastic deformation of composites is deeply dependent on thermal residual stress and strain rate. The two parameters' influence the biaxial yield response of polymer-matrix composites is discussed in this section. In these cases, circular fiber-reinforced composites with 0.515 fiber volume fraction are considered. Thermal expansion coefficients of the glass fiber and matrix can be seen in Table 2. The thermal residual stress calculation can be seen in reference [30].

#### 4.2.1. Thermal Residual Stress Influence on Yield Surface

**Figure 10.**
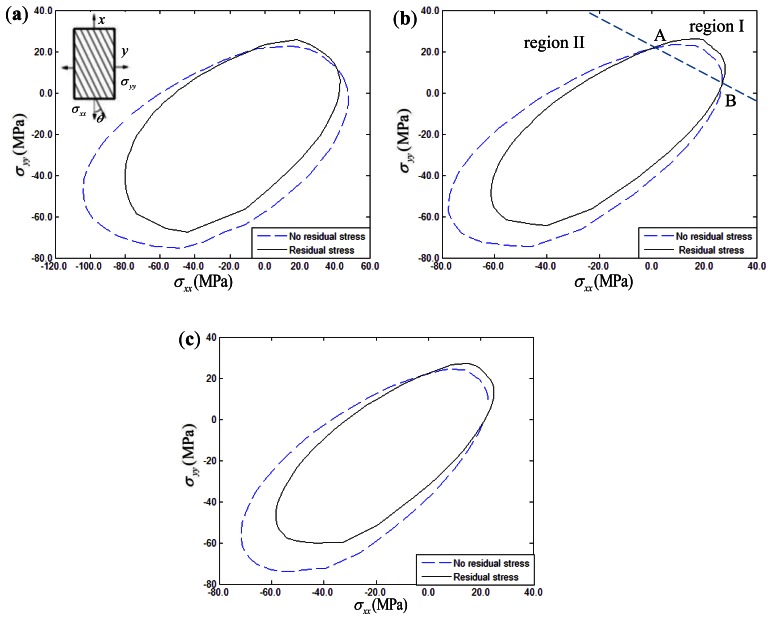
Thermal residual stress influence on σ*_xx_* – σ*_yy_* yield surface of composites: (**a**) 15° fiber-reinforced composites; (**b**) 30° fiber-reinforced composites; (**c**) 45° fiber-reinforced composites.

**Figure 11.**
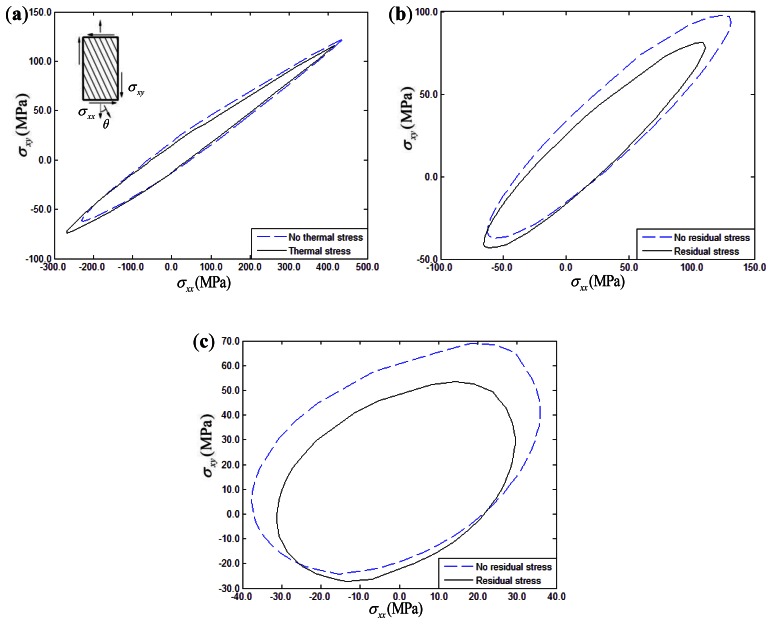
Thermal residual stress influence on the σ*_xx_* – σ*_xy_* yield surface of composites: (**a**) 15° fiber-reinforced composites; (**b**) 30° fiber-reinforced composites; (**c**) 45° fiber-reinforced composites.

For biaxial loading under constant strain rate conditions (*ε̇_xx_* = *ε̇_yy_* = 10^–5^ or *ε̇_xx_* = *ε̇_xy_* = 10^–5^), thermal residual stress effects on the yield surface of 15°, 30°, 45° fiber-reinforced composites are discussed. For comparison purposes, the yield surface of composites disregarding the thermal stress effect is also shown in the corresponding figures. Figures 10 and 11 show the yield surface in the σ*_xx_* – σ*_yy_* and σ*_xx_* – σ*_xy_* stress planes, respectively. The temperature drop was assumed to be 150 °C in the simulation examples. Taking Figure 10(b) as an example, it can be seen that the yield surfaces of composites with thermal stress and without thermal stress have two intersections, namely intersection A and B.

The region above the line A-B is defined as region I, while the other one is defined as region II. From the Figures 10 and 11, it can be seen that the biaxial yield response of composites had a characteristic two-region deformation behavior. However, the thermal residual stress effects on the yield surface of composites exhibits opposite variation between the σ*_xx_* – σ*_yy_* and σ*_xx_* – σ*_xy_* stress planes. In details, the biaxial yield strength of composites in the σ*_xx_* – σ*_yy_* stress plane tends to be increased to a certain extent when the thermal residual stress is taken into account in region I, while the thermal residual stress tends to decrease the yield strength in region II. However, it is interesting to mention that the opposite variation can be found in the σ*_xx_* – σ*_xy_* stress plane. The yield strength intends to decrease when the thermal residual stress is taken into account in region I. However, thermal residual stress intends to increase the biaxial yield strength when the load is located in region II. Furthermore, comparing with other σ*_xx_* – σ*_xy_* stress conditions, the thermal residual stress effects on the biaxial yield response for 15° fiber-reinforced composites can be ignored.

#### 4.2.2. Strain Rate Influence on Yield Surface

**Figure 12.**
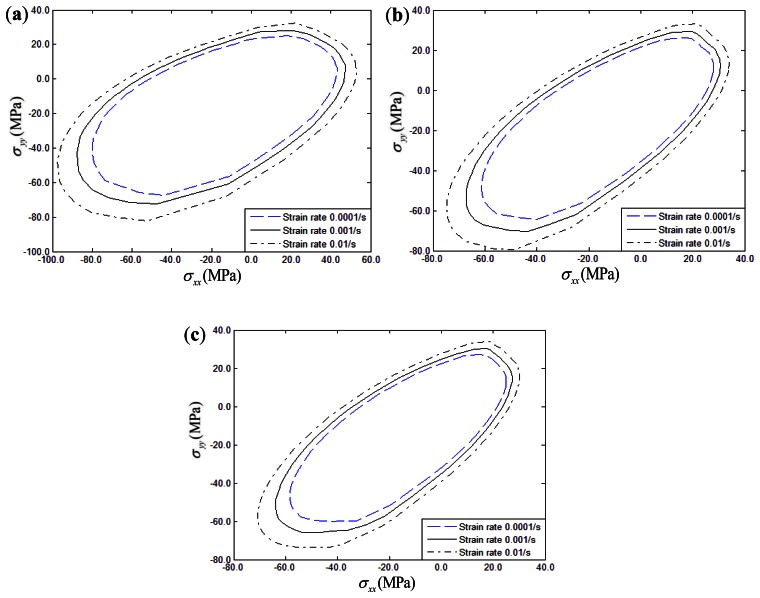
Strain rate influence on σ*_xx_* – σ*_yy_* yield surface of composites: (**a**) 15° fiber-reinforced composites; (**b**) 30° fiber-reinforced composites; (**c**) 45° fiber-reinforced composites.

**Figure 13.**
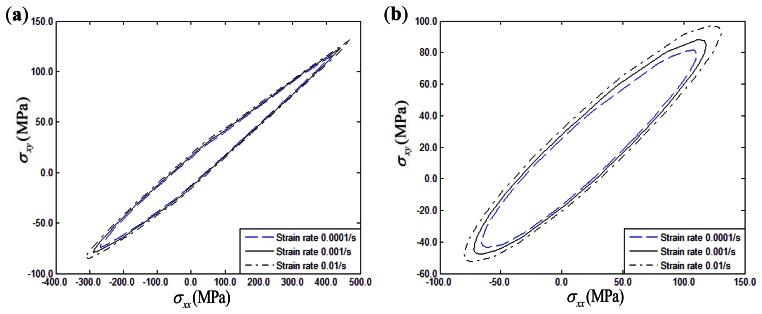

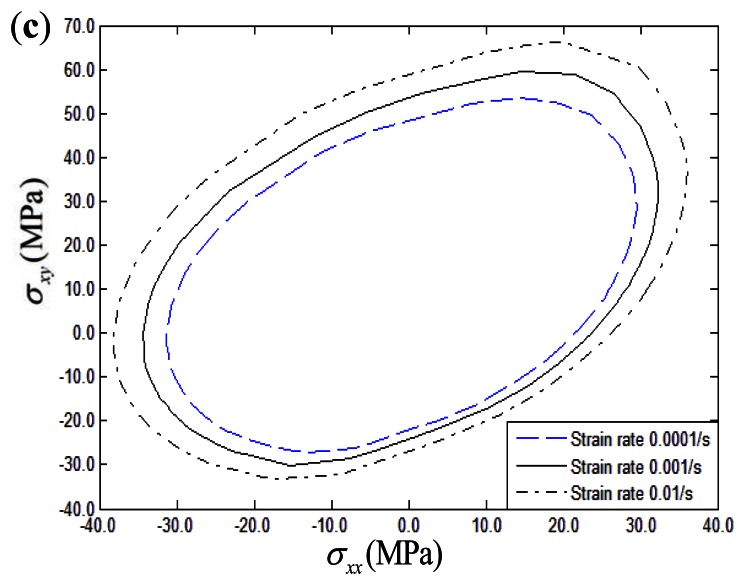
Strain rate influence on σ*_xx_* – σ*_xy_* yield surface of composites: (**a**) 15° fiber-reinforced composites; (**b**) 30° fiber-reinforced composites; (**c**) 45° fiber-reinforced composites.

The effects of a strain rate range of 0.0001/s to 0.01/s on the biaxial yield strength of fiber-reinforced composites with thermal residual stress under σ*_xx_* – σ*_yy_* and σ*_xx_* – σ*_xy_* conditions can be seen in Figures 12 and 13, respectively. Three different fiber off-axis angles (15°, 30°, 45°) are discussed. From the figures, it can be found that the biaxial yield strength exhibits a significantly rate-dependence in the σ*_xx_* – σ*_yy_* and σ*_xx_* – σ*_xy_* stress planes similar to the uniaxial loading conditions. Increasing the strain rate will increase the biaxial yield strength of polymer-matrix composites. Furthermore, strain rate effects on the biaxial yield surface can be hardly discerned if the fiber off-axial angle is 15° in the σ*_xx_* – σ*_xy_* stress plane.

## 5. Conclusions

The Generalized Method of Cells can be used to predict the nonlinear stress-strain of metal-matrix composites, polymer-matrix composites and ceramic-matrix composites through incorporating different viscoplastic models. In this paper, the method has been used to investigate the thermal residual stress and strain rate influence on the biaxial yield responses of polymer-matrix composites with different fiber off-axis angles. In the σ*_xx_* – σ*_yy_* stress plane, thermal residual stress tends to increase and decrease the biaxial yield strength of composites in region I and region II, respectively. However, the law for the σ*_xx_* – σ*_xy_* stress plane, which is influenced by thermal residual stress, shows the opposite variation. In addition, increasing strain rate tends to increase the yield strength of composites in both the σ*_xx_* – σ*_yy_* and σ*_xx_* – σ*_xy_* stress planes, which is unrelated to the fiber off-axis angle.

## Figures and Tables

**Table 1.** Sensitivity and initial wavelength error analysis of FBGs sensor.

**Loading condition**	**Theoretical value* λ*_0_/nm**	**Measured value* λ*_0_/nm**	**Absolute error *λ*_0_/nm**	**Sensitivity /(pm/με)**	**Sensitivity relative error**	**Related coefficient**
loading	1560.2390	1560.2403	0.0013	1.194	0.50%	0.9959
unloading	1560.2390	1560.2413	0.0023	1.158	3.50%	0.9948

**Table 2.** Material parameters of the glass fiber and polymer matrix.

**Material**	**Elastic modulus *E*/GPa**	**Poisson's ratio *v***	***n***	*α*_1_	*α*_0_	*Z*_0_/**Gpa**	*Z*_1_/**Gpa**	$D_{0}^{-1}$/**s**	***q***	*α*
glass fiber	71.42	0.2	-	-	-	-	-	-	-	5 × 10^−6^
Polymer	3.3	0.22	0.63	0.104	0.184	0.391	0.803	10^-6^	168.5	25 × 10^−6^
